# Evaluating the Benefits of Transition to Home Palliative Care: Pharmacological Prescriptions, Social, and Psychological Support Post-Referral

**DOI:** 10.1177/21501319241285340

**Published:** 2024-11-28

**Authors:** Hugo Ribeiro, João Rocha-Neves, Marília Dourado, José Paulo Andrade

**Affiliations:** 1 Community Palliative Care Team Gaia, Local Health Unit of Gaia and Espinho, Vila Nova de Gaia, Portugal; 2 Faculty of Medicine of University of Coimbra, Coimbra, Portugal; 3 Faculty of Medicine of University of Porto, Porto, Portugal; 4 Center for Innovative Biomedicine and Biotechnology (CIBB), Coimbra, Portugal; 5 Coimbra Institute for Clinical and Biomedical Research (iCBR)-Group of Environment, Genetics and Oncobiology (CIMAGO), University of Coimbra (FMUC), Coimbra, Portugal; 6 RISE@Health, University of Porto, Porto, Portugal

**Keywords:** terminal care, referral and consultation, home care services, patient care team, community networks

## Abstract

**Introduction/Objectives:**

Community palliative care support teams specialize in providing at-home care under referral criteria distinct from hospital teams, focusing on functional status, limited benefit from continued hospital specialty care, and increased clinical complexity. This study aimed to assess the quality of referrals and the transition of care to a specialized community palliative care team, emphasizing adherence to established referral criteria.

**Methods:**

An observational, longitudinal, retrospective cohort study evaluated clinical health records of patients who received palliative care from a community team in 2023. We evaluated prior multidisciplinary follow-up, basic social support, medication changes made during the team’s first consultation, and recurrences to hospital emergency care. The data was collected from the patients’ clinical files.

**Results:**

The patient cohort had a mean age of 80.7 years (±11.92), ranging from 31 to 103 years. Males comprised 56.3% of the participants, with a median follow-up time of 32 days. Primary diagnoses included cancer (50%), end-stage organ failure (26%), and neurodegenerative disease (24%). Among the deceased, 85.6% died at home. Patients showed marked changes in psychological support and social rights, as we observed an improvement from 6.8% to 100% (*P* = .0011) and from 47.1% to 100% (*P* = .01) in these supports, respectively. We also observed significant changes in pharmacotherapeutic plans, regardless of the referring team. The study observed significant difficulty in recognizing referral criteria for specialized palliative care and defining clinical complexity. Referrals were often delayed, particularly for those previously under hospital care (*P* = .001). Increased prescription of pro re nata medications significantly correlated with home deaths (*P* = .021).

**Conclusions:**

Most complex patients can be effectively monitored and die at home, reserving hospital deaths for exceptional cases requiring interventions not possible at home or due to significant caregiver burden. There was no difference in the biopsychosocial approach of patients previously followed by other teams, including hospital palliative care teams, which suggests very different approaches.

## Introduction and Objectives

According to the World Health Organization (WHO), annualy, an estimated 4.4 million people need palliative care.^1^

In western Europe countries, recommendations advise a palliative home care specialized team for every 100 000 inhabitants, with at least 2 medical doctors, 3 nurses, 1 social worker, and 2 psychologist.^2^ In a study carried out in 51 European countries in 2020, the authors concluded that specialized service provision increased throughout Europe, yet ratios per 100 000 inhabitants fell below 50% of the European Association for Palliative Care (EAPC) recommendations.^3^

To accommodate the preference of end-of-life patients to die at home, it is essential to have a team specialized in palliative care, which is only necessary for up to 30% of patients due to clinical complexities.^4^ Furthermore, robust social support is crucial, providing technical and financial support and ensuring rights for family members and caregivers, alongside solid psychological aid to prevent caregiver burnout and mitigate the psycho-affective impact of witnessing a loved one’s suffering.^5^

Within the scope of medical follow-up, it is necessary to ensure therapeutic adequacy to the patient’s current situation, efficiency in controlling symptoms, and guarantee of pro re nata (PRN) medication at home for administration if there is a sudden worsening of the general or symptomatic condition.^5^

The EAPC recommendations for minimum ratios for community palliative care teams are not based on robust scientific evidence that measures added health value. There is a lack of knowledge about the potential effects of a multidisciplinary team on the health of the patient and their family. As far as we know, there are no studies that evaluate health care indicators that cover the different sectors of palliative care, particularly focused on the transition of care, a moment that is intended to be as smooth as possible for the patient and their family.

In this study, some of the indicators of palliative care quality were assessed, such as the effectiveness and efficiency of the care provided, the quickness of access to a multidisciplinary palliative care team and the change made when a patient is integrated for follow-up by the team. This knowledge could help to redefine the ratios of community teams and establish indicators of quantity and quality of care provided.

Therefore, the main objective was to evaluate the change made in the first consultation of a community palliative care team in the biopsychosocial aspects of patients and their families, comparing the previous approach to the patient and family (family health teams, other hospital medical specialties, and also hospital palliative care teams) with the multidimensional and multidisciplinary approach carried out by the team that collaborated in the study. Our secondary objectives were: (a) to assess the celerity and adequacy of referral to the team considering the referral criteria adopted, and possible consequences; (b) to compare prescription practices between home, hospital and other palliative care teams; (c) to assess differences in family care and psychosocial support provided depending on the type of team providing care; (d) to assess the main determinants of hospital death for patients followed by a community palliative care team; and (e) to analyse the reasons for using the emergency department and assess whether these could have been prevented if there had been an earlier referral.

## Methods

This is an observational, retrospective cohort study without intervention. Between January and December 2023, all the patients who were referred to our specialized palliative care community support team in the northern region of Portugal were included in this study. Of the 450 patients referred to the team during this period, 127 patients were not included in the study due to not meeting the inclusion and referral criteria (these patients were not followed directly by the team, they were followed through consultation with family doctors). Thus, the study sample consisted of 323 patients, which were all patients directly followed by the team during 2023.

The study protocol was approved by the local Ethics Committee and respects the Declaration of Helsinki. Patient informed consent was handled accordingly, and all data processing was anonymous.

The team that participated in this study had 4 medical doctors, 5 nurses, a social worker, and a psychologist. It works in a municipality of the north of Portugal and has a coverage area of 43 km^2^ with 180 000 inhabitants. The team operates from 8:00 am to 7:00 pm, Monday through Friday. All patients and families can get in direct contact with all sectors of the team (doctor, nurse, social worker, and psychologist) during opening hours. And all patients have a daily or twice-daily nursing teleconsultation to check their current status and organize priority in-person consultations at home.

The team has the following referral criteria: (a) patient has a high clinically complex disease; (b) palliative performance scale <60%^6^; (c) patient has a caregiver, ideally for 24/24 períod; and (d) difficult in symptom control due to its severity and/or symptoms with great daily fluctuation.

The data source/ measurement was the patient’s clinical file.

We evaluated the following variables in the study patients: gender, age, reference date, date of team integration, date and reason for team discharge, main pathology, the unit of origin, fulfillment of the 4 referral criteria individually, assessment of basic social support considering only the multipurpose certificate and the dependency supplement, basic rights for any patient with an advanced chronic illness, prior psychological support (yes or no, for the patient and/or caregiver), changes to fixed medication, and PRN medication in the first consultation of the palliative care team at home. It was also assessed the place of death, the guidance given to the patient in the last consultation when they did not die at home, follow-up time by the team (time elapsed from the first consultation until the patient’s death or clinical discharge), and all visits to the emergency department, particularly the reasons and timing of these recurrences.

Statistical analysis was done using SPSS (Statistical Package for the Social Sciences software version 28 for Windows). Statistical analysis descriptive measures were used: categorical variables with absolute and relative frequencies and continuous variables with mean and respective standard deviation. For inferential statistics, the Chi-square test, the Kruskal-Wallis test, the Anova 1-Way test, Mann-Whitney *U* test, and Student’s *T* test. The Chi-square assumption that there should be no more than 20% of cells with expected frequencies less than 5 was analyzed. When this assumption was not satisfied, the Chi-square test was used using the Monte Carlo simulation. Differences were analyzed with the support of standardized adjusted residuals. The homogeneity of variances was analyzed using Levene’s test. The significance level to reject the null hypothesis was set at α ≤ .05.

## Results

The final sample from which the results are presented was made up of 323 patients. The average age of the patients was 80.7 years, varying between a minimum of 31 and a maximum of 103 years. The majority were male (56.3%). The median follow-up time was 15 interquartile range [5-39] days.

Regarding the origin of referrals, 118 patients were referred by hospital palliative care teams, nursing homes, and long-term care units referred 46 patients, and 100 patients were referred by family doctors. Finally, 59 patients were referred from various medical specialties, including 33 from oncology.

As shown in Table 1, around 50% of these patients had cancer as their main diagnosis, 26% had an organ failure, and 24% had a neurodegenerative disease. Table 1 also shows the number of patients referred by the referencing unit. No statistically significant relationship existed between the place of death and the referencing unit, except for oncological disease, in which we observed a greater referral by other hospital specialties, given that 33 of these 59 patients were referred by oncology.

**Table 1.** Main Diseases of Patients Followed by the Home Palliative Care Team.

	N	%
Total of patients	**323**	**100%**
Main pathologies		
Dementia	59	18.3
Neurodegenerative disease	14	4.3
Heart failure	38	11.8
Renal failure	11	3.4
Respiratory failure	7	2.2
Oncological disease	162	50.2
Frailty	30	9.3
Hepatic failure	2	0.6
Referencing unit		
Hospital Palliative Care	118	36.50
Family Physician	100	31.00
Other medical specialties	59	18.30
Nursing home	46	14.20

Abbreviations: %, percentage; N, number of patients.

Regarding the referral criteria for the team, in 12.70% of patients there was no difficulty in symptomatic control, 4% of patients had a functionality measured by the Palliative Performance Scale (PPS) higher than the team’s referral criteria (PPS less than 60%), 7.4% of patients did not have a guaranteed caregiver for 24 h a day, 52.9% of patients did not have basic social support and 93.2% did not have psychological support before the start of follow-up by the team.

The median time interval between referral and the first consultation was 24 h (interquartile range [2-56]).

Of the 323 patients followed in 2023, 195 died (60.4%). Regarding the places of death of these patients, we observed that 73.8% of patients died at home and 10.3% died in nursing homes, meaning that 84.1% of patients died in their current residence. We also observed that 14.4% of patients died in hospital palliative care inpatient unit and 1.5% died in a palliative care unit.

The team referred 143 patients at a given point in their follow-up to other units (44.3%). Family doctors received more referrals (19.2%), followed by nursing homes (10.5%), meeting the need for joint efforts, particularly in treating wounds and skin ulcers. The 5 patients referred to palliative care units (1.5%) met the caregiver rest criterion.^7–9^

All patients referred to the emergency service and who died in hospital (n = 14, 4.3%) and to hospital admission for palliative care inpatient unit (n = 22, 6.8%) met criteria of caregiver exhaustion and/or difficulty managing medication of high complexity at home, particularly in situations in the last days of life (n = 33). The 5 patients (1.5%) referred to the hospital palliative care team for follow-up in an external consultation were patients who still had functionality that allowed them to go to the appointment without compromising their well-being and still benefited from follow-up by other hospital specialties. One patient was referred to another community team because he had changed his residence, leaving him outside the team’s area of influence.

All 37 recurrences to the Emergency Department were analyzed. Of the 14 patients who died after this event, 12 (85.7%) could not have been avoidable by extending the team’s hours of coverage. On the other hand, of the remaining 23 recurrences to the Emergency Department of patients followed by the team during the year 2023, 18 (78.3%) were while the team was on duty. In 13 of these cases (72.2%), the team made the referral request. Only 1 (4%) had the potential to be avoidable in the remaining 5 recurrences with greater time coverage by the team.

**Figure 1.**
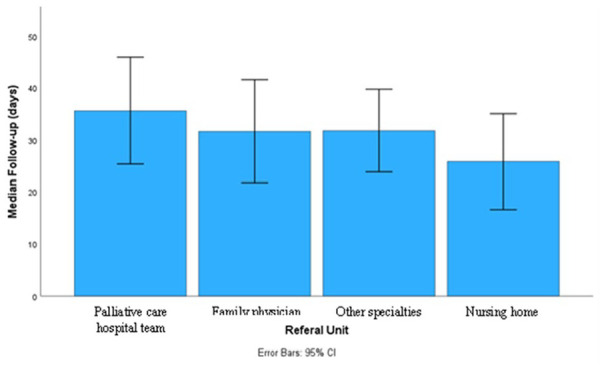
Median follow-up time by the team and referral unit. Median follow-up (days) and standard deviation of patients came from palliative care hospital team: 39 ± 39.25. Median follow-up (days) and standard deviation of patients came from family physicians: 31.67 ± 49.81. Median follow-up (days) and standard deviation of patients came from other specialties: 31.78 ± 30.42. Median follow-up (days) and standard deviation of patients came from nursing homes: 25.85 ± 31.17. %, percentage.

We also observed that the follow-up time was longer in patients who came home from palliative care units (median of 39 [5-39.25] days) and shorter in patients who were in nursing homes (median of 11 [2-45.25] days), although the differences were not significant (*P* = .220). Figure 1 shows the follow-up time in different patients’ actual residences.

The time that elapsed between the first consultation with our team and home death was variable, with a cutoff point, in terms of follow-up days, of 9.5 days. No statistically significant relationship existed between the place of death and the referencing unit.

Regarding the number of changes made in the first consultation with our team, both in the fixed medication and in the PRN medication, we observed an average of 3.78 changes in fixed medications (minimum of 0 and maximum of 13) and 3.01 in PRN medications (minimum of 0 and maximum of 8).

Regarding the changes made to the medication at the first consultation and their relationship with the place of death, there were no significant differences concerning the fixed medication. However, there were significant differences concerning the PRN medication that were significantly higher in patients who died at home (*P* = .021; Table 2).

**Table 2.** Place of Death and Changes to Medication at First Consultation.

	PCU		Hospital		NH		Home		*P*
	A	SD	A	SD	A	SD	A	SD	
Fixed medication changes at the first appointment	3.00	2.00	3.82	2.46	3.95	2.28	4.56	2.53	.320
**PRN medication changes at First appointment**	**4.00**	**2.64**	**3.36**	**1.54**	**2.35**	**1.26**	**3.33**	**1.56**	.**021**

Abbreviations: A, average; NH, nursing home; *P*, significance; PCU, palliative care unit; PRN: pro re nata; SD, standard deviation.

Highlighted in bold the main differences (statistically significant).

For patients who died at home, the cutoff point for changes to fixed medication at the first consultation is 3.5 changes, and the cutoff point for changes in PRN medication is 2.5 changes.

Table 3 shows the number of patients meeting the referral criteria and referencing unit. Regarding symptom assessment, a significantly higher proportion (*P* = .018) of patients with poorly controlled symptoms at the first consultation come from other medical specialties (96.6%). Patients without adequate Palliative Performance Scale (PPS) are significantly more likely (*P* = .03) to be referred by family doctors (8%).

**Table 3.** Number of Patients Meeting the Referral Criteria for the Team and Referencing Unit.

		PCHT	FP	Other	NH	*p*
Difficulty in symptom control						.**018**
No	N	15	20	2	4	
	%	12.7	20.0	3.4	8.7	
Yes	N	103	80	57	42	
	%	87.3	80.0	96.6	91.3	
PPS						.**031**
No	N	5	8	0	0	
	%	4.2	8.0	0.0	0.0	
Yes	N	113	92	59	46	
	%	95.8	92.0	100.0	100.0	
Caregiver 24 h/24 h						.124
No	N	9	11	4	0	
	%	7.6	11.0	6.8	0.0	
Yes	N	109	89	55	46	
	%	92.4	89.0	93.2	100.0	
Basic social support						.**001**
No	N	69	56	43	3	
	%	58.5	56.0	72.9	6.5	
Yes	N	49	44	16	43	
	%	41.5	44.0	27.1	93.5	
Psychological support						.**011**
No	N	110	97	58	36	
	%	93.2	97.0	98.3	78.3	
Yes	N	8	3	1	10	
	%	6.8	3.0	1.7	21.7	

Abbreviations: %, percentage; A, average; FP, family physician; NH, nursing home; Other, other medical specialties; *P*, significance; PCHT, palliative care hospital team; PPS, Palliative Performance Scale; SD, standard deviation.

Highlighted in bold the main differences (statistically significant).

Regarding basic social support, patients who came from nursing homes are most likely to have these requirements (93.5%). On the other hand, only 72.9% of patients referred from other medical specialties had basic social support, which is significant (*P* < .001). There is a significantly higher proportion of patients (*P* < .001) with adequate psychological support among patients referred by nursing homes (21.7%). However, this criterion is clearly least met when referring to the team.

Table 4 shows the place of death and orientation for other services and the referencing unit. There is a significantly higher proportion of patients (*P* < .001) whose place of death was in the nursing home, with referrals coming from the nursing home itself (84.2%). There is a significantly higher proportion of patients referred to palliative care units in patients referred by the hospital palliative care team (9.3%; *P* = .001) and with guidance for palliative care hospital team in patients coming from other medical specialties (38.9%; *P* = .001).

**Table 4.** Place of Death and Orientation for Other Services and Referencing Unit.

		PCHT	FP	Other	NH	*P*
Place of death						.**001**
Palliative Care Unit	N	2	0	1	0	
	%	2.3	0.0	2.3	0.0	
Hospital	N	12	7	7	2	
	%	14.0	14.9	16.3	10.5	
Nursing home	N	3	1	0	16	
	%	3.5	2.1	0.0	84.2	
Home	N	69	39	35	1	
	%	80.2	83.0	81.4	5.3	
Orientation for other services						.**001**
Palliative Care Unit	N	4	0	1	0	
	%	9.3	0.0	5.6	0.0	
Emergency departament	N	3	6	4	1	
	%	7.0	11.5	22.2	3.4	
Palliative care nursery	N	9	5	7	1	
	%	20.9	9.6	38.9	3.4	
Family physician	N	20	37	5	0	
	%	46.5	71.2	27.8	0.0	
Nursing home	N	3	3	1	27	
	%	7.0	5.8	5.6	93.1	
Palliative care hospital team	N	4	1	0	0	
	%	9.3	1.9	0.0	0.0	

Abbreviations: %, percentage; A, average; FP, family physician; NH, nursing home; Other, other medical specialties; *P*, significance; PCHT, palliative care hospital team; SD, standard deviation.

Highlighted in bold the main differences (statistically significant).

Patients referred by a family doctor were more likely to be referred back to a family doctor (71.2%), patients referred by nursing homes were more likely to be referred back to nursing home teams (93.1%), and the number of patients referred to the hospital palliative care team were more likely to be referred from that team (9.3%; *P* < .001).

Regarding changes in PRN medication in the first consultation, the average change is significantly lower (*P* < .001) in patients referred by nursing homes (2.17) when compared to the changes made to patients referred by the hospital palliative care team (3.22), by family doctors (2.99) or other medical specialties (3.25). No statistically significant differences were related to lack of symptomatic control at the first consultation and place of death (*P* = 1.000).

The existence of a 24/7 caregiver and the place of death was statistically significant (*P* < .001). There is a significantly higher proportion of patients with a 24-h caregiver and death at home and of patients without a 24-h caregiver and death in hospital (67.9%).

Regarding the guidance the team gave, there is a significantly higher proportion of patients with a 24/24-h caregiver to be followed by a family doctor (48%) or a nursing home (26.8%). There is a significantly higher proportion of patients without 24/24-h caregivers referred to the emergency service (33.3%) or for palliative care hospitalization (46.7%), as shown in Table 5.

**Table 5.** Guidance and Caregiver 24 h/24 h.

Orientation for other services	Caregiver 24 h/24 h			
	No		Yes	
	N	%	N	%
Palliative Care Unit	1	6.7	4	3.1
Emergency department	5	33.3	9	7.1
Palliative Care inpatient unit	7	46.7	15	11.8
Family doctor	1	6.7	61	48.0
Nursing home	0	0.0	34	26.8
Palliative care team extern consult	1	6.7	4	3.1

Abbreviations: %, percentage; N, number of patients.

The days of follow-up were significantly lower in frailty (average of 17.63 days) than in oncological disease (average of 31.27 days), dementia (average of 47.8 days), and renal failure (average of 40.27 days), and the days of follow-up were significantly lower in heart failure (average of 26.18 days) than in dementia (*P* = .021).

## Discussion

It is desirable that the transition of care is felt by the patient and their family in a slight way. In other studies, we found several differences in the biopsychosocial approach in the transition from hospital care to community care.^10-12^ However, other studies did not compare the transition to community palliative care specialized teams but analyzed the transition to community general practitioners and family medicine teams.

The community palliative care team presents several high-performance indicators, including adequate control of symptoms and mitigation of suffering in the biopsychosocial aspects and the speed of response to referral with a median below 24 h, which follows the defining principles of the response in palliative medicine.^13^

According to previous literature, it would be expected that there would be between 2477 and 3205 patients referred by year to palliative care specialized teams in the intervention area of the team that collaborated in this study.^14^ We estimate that this team could have between 400 and 500 patients referred by year. In the year under study (2023) this team had 450 referrals.

The demographic assessment of the study population and the average follow-up (32 days) is in line with what has been reported in other studies for home palliative care teams, which end up monitoring more terminally ill patients.^15-17^ However, it would be expected that, according to the team’s referral criteria, they could present a median follow-up time that is more appropriate to the life expectancy of the terminally ill patient (3-6 months of life).^18^

The follow-up time was longer in patients who came home from palliative care units and shorter in patients who were in nursing homes. This difference demonstrates that most patients referred to palliative care units are sent due to caregiver burden, whereas those referred from nursing homes require intermittent support from the team.

Regarding the primary diseases of the patients, oncological diseases account for half of the patients, followed by organ failure and neurodegenerative diseases. This number of patients with non-oncological diagnoses that limit prognosis is notably high compared to experiences in other locations.^19,10^ However, this number aligns with results from several international scientific societies.^21,22^

The team demonstrates considerable flexibility in assessing cases referred to them. This becomes particularly evident when considering the large number of patients who lack adequate basic social support (52.9%), lack of a caregiver (7.4% of referred patients), or guaranteed psychological support for themselves and/or their families (93.2%) at the first consultation. All patients had their basic social needs and psychological support guaranteed during this team’s first 2 weeks of follow-up, which represents an improvement in a multidimensional and multidisciplinary approach that may be easier to implement in the community, at patients and their families homes.

In other studies, we can identify that the choice of the preferred place of death has corresponded to the actual place of death in very different percentages (25%-50%).^23,24^ A previous investigation by this team^25^ reported that 74% of patients (n = 121) died at home, with 82.6% having made this wish previously. In this study, we conclude that 85.6% of the patients died at their current residence.

Longer follow-up times were associated with an increased likelihood of death occurring at home. The importance of long-term follow-up for gaining confidence and training in-home care is evident. A significantly higher proportion of patients with poorly controlled symptoms at the first consultation come from other medical specialties, and patients without adequate PPS are most likely to be referred by family doctors. Therefore, the team must invest in promoting and training other teams and reference groups to ensure earlier referrals. This data is also in line with what we found from other studies, that identify the ideal moment for referral between the final 3 and 12 months of life.^26,27^

The data highlighted that changes to “as needed” medications at the first consultation were significantly greater for patients who eventually died at home. This data reinforces the need for continuous work with caregivers to teach medication management, particularly in unexpected situations.

Patients referred from a family doctor are more likely to be referred back to a family doctor (71.2%), patients referred by nursing homes are more likely to be referred back to nursing home teams (93.1%), and the number of patients referred to the hospital palliative care team were more likely to be referred from that team (9.3%). These data point to the intermittent need for intervention by a team specialized in palliative care and not on a continuous basis, making it a good idea to adjust the patient’s circuit according to their individual needs, as it was pointed out by Lucket et al.^28^ This continuous adaptation of the referencing model is in accordance with the literature^28^ and also some operational guidelines^29^ that foresee that support models in the area of specialized palliative care are expected to be individualized and flexible according to local experiences and needs.

There is a significantly higher proportion of patients with a 24-h caregiver and death at home and of patients without a 24-h caregiver and death in hospital. The fundamental role of the caregiver for a safe and peaceful death at home has already been identified by several studies, as well as their needs for social and differentiated support in palliative care.^30-32^ This study reinforces the need to guarantee the existence and continuous presence of a caregiver so that the desired death occurs at home.

## Study Limitations

The study’s main limitation is the heterogeneity of the sample, which does not consider prior follow-up to adequately control the quality of the procedures and evaluate the results. As this was a retrospective study, dependent on the evaluation of records in the clinical process, it was not possible to evaluate justifications for individual clinical decision-making. Multivariable analysis was also conditioned due to the study’s retrospective design, the heterogeneity of the data, and the symptom-centered approach to the disease, which constrained the ability to control for multiple confounders simultaneously. Another significant limitation is that the study is single-center, limiting the generability of the processes. Important variables, such as patient-reported outcomes and safety, were also not evaluated. Data from a historical cohort was also not obtained.

## Conclusions

With the data obtained, we verified that this team is making a major change in follow-up. Patients and their caregivers received psychological support and had their most basic social rights guaranteed, something that a large percentage of patients did not have before the team’s care. They also had a very significant change in their pharmacotherapeutic plan, progressing from a fixed medication to a PRN medication. An individualized, integrated care plan was established, with consultation from their family health team. Therefore, this team seems to provide an adequate response to its area of intervention, which can be considered an example of differentiated ratios and indicators in home palliative care.

The data collected should lead us to some reflections: teams may not have enough human resources for their needs, palliative medicine training does not appear identical, and referral to the community team is being done too late. Regarding this last issue, it seems that patients are being referred too late to this team, particularly those being followed by hospital teams, including patients referred by hospital palliative care teams.

One of the most interesting data from this study is related to recurrences to the emergency room. It is a declared objective by government entities to ensure a 24 h/24 h response from community teams. However, as noted, there are very few situations in which this team’s extended evening hours could have prevented a recurrence of the emergency room. This data is very relevant for the organizational strategy to be followed for these teams and should not be evaluated separately in all other aspects of biopsychosocial follow-up, from the increase in the exacerbation prevention strategy with PRN medication to appropriate psychosocial support for patients and caregivers.

The institution of PRN medication, that is, the strategy of teaching the caregiver to react to more or less unexpected situations, is significantly related to death at home. Evidence evaluating pro re nata medication is scarce, but several studies point to the need to introduce it in the elderly, frail patients with complex clinical needs, with this prescription being more common towards the end of life.^33^

These findings underscore the need for continuous training for referring teams and the integration of palliative care principles across all levels of patient care. The emergency room recurrences and the strategic importance of PRN medication further emphasize the necessity for a well-resourced, and timely palliative care approach.

Overall, this study contributes valuable insights into the optimization of palliative care services, advocating for earlier referrals and better-coordinated care transitions to improve patient outcomes and align with best practices in palliative medicine.

## Future Perspectives

In future studies, we will consider comparing outcomes related to the patient’s perspective and outcomes of technical-scientific quality, just as we would like to compare data with other similar teams, in identical regions from the point of view of health and social support.

We would like to carry out prospective, longitudinal studies to adequately evaluate procedures and results, according to individualized decisions, before and after the start of follow-up by the team.
